# Cardiac Failure as an Unusual Presentation in a Patient with History of Amyotrophic Lateral Sclerosis

**DOI:** 10.1155/2014/986139

**Published:** 2014-07-15

**Authors:** Mohammad Hasan Namazi, Isa Khaheshi, Habib Haybar, Shooka Esmaeeli

**Affiliations:** ^1^Cardiovascular Research Center, Modarres Hospital, Shahid Beheshti University of Medical Sciences, Tehran, Iran; ^2^Cardiovascular Research Center, Ahvaz Jundishapur University of Medical Sciences, Ahvaz, Iran; ^3^Students Scientific Research Center (SSRC), Tehran University of Medical Sciences (TUMS), Tehran, Iran

## Abstract

Amyotrophic lateral sclerosis (ALS) is the most well-known form of motor neuron diseases in which both upper and lower motor neurons are involved in this disease. We presented an unusual case of ALS whom had presented with chief complaint of dyspnea. Cardiac failure was diagnosed at the final stage of the ALS disease. The pathogenetic mechanism leading to an elevated occurrence of cardiomyopathy in ALS is not comprehensible. Dilated cardiomyopathy has been explained in some previous studies. Based on the collected data, it was hypothesized that cardiomyopathy is underdiagnosed in the ALS population, probably because symptoms are masqueraded as a result of the patients' disability. It was suggested that in all motor neuron diseases a serial cardiological evaluation should be executed, including annual echocardiography.

## 1. Introduction

Amyotrophic lateral sclerosis (ALS) is the most well-known form of motor neuron diseases in which both upper and lower motor neuron are involved in it. We presented an unusual case of ALS that presented with the chief complaint of dyspnea. Cardiac failure was diagnosed at the final stage of the ALS disease [1, 2]. Cardiac involvement has been rarely illustrated as part of the motor neuron diseases among literature [3].

Dilated cardiomyopathy has been explained in some previous studies [3]. Based on the collected data, it was hypothesized that cardiomyopathy is underdiagnosed in the ALS population, probably because symptoms are masqueraded as a result of the patients' disability. It is suggested that in all motor neuron diseases a serial cardiologic evaluation should be executed, including annual echocardiography.

## 2. Case Presentation

A 72-year-old man with history of amyotrophic lateral sclerosis (ALS) from 3 years ago presented in the emergency unit with complaint of dyspnea exacerbation from New York Heart Association (NYHA) functional class II to IV, orthopnea, and paroxysmal nocturnal dyspnea from 6 weeks before presentation. He had a history of stent angioplasty on LAD and RCA which was performed for him 4 years ago. His echocardiography findings after stent angioplasty revealed ejection fraction of 50%. After that, he was in stable condition, until progressive weakness and atrophy of left upper limb started and involved right upper limb and then lower limbs gradually, and diagnosis of ALS was established due to clinical presentation, electromyography (EMG) findings, and ruling out of the other similar motor neuron disease. Riluzole was prescribed for him but it was not tolerated due to significant side effects including nausea, headache, and dizziness. Recently, he has had dysphagia to liquids.

On physical examination, blood pressure was 105/70 mmHg, heart rate was 92/min, respiratory rate was 20, and body temperature was 36.9°C. On chest auscultation, there were fine rales on the base of both lung fields, and grade II systolic murmur on apex and left sternal border were heard.

**Figure 1:**
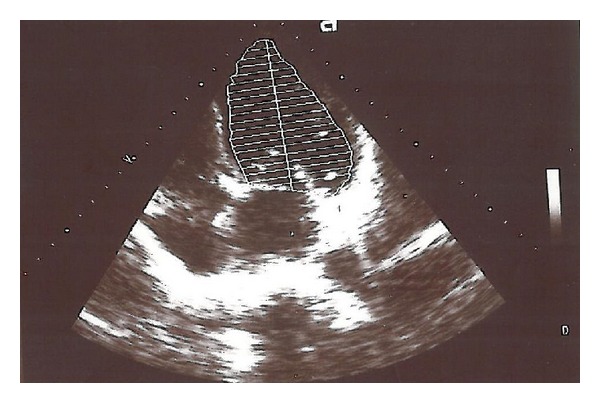
Transthoracic echocardiography revealed global hypokinesia of the LV with left ventricular ejection fraction (EF) of 20% which was measured by biplane Simpson method. The three-year-earlier echocardiography had showed ejection fraction of 50%.

The new echocardiography was done for him which showed ejection fraction of 20% with global hypokinesia (Figure 1 and Table 1); myocardial perfusion scan was normal which is highly predictive for the absence of CAD in the setting of heart failure and left ventricular dysfunction. He had no history of recent viral or bacterial infections. Serial ECGs showed no significant ST-T changes.

Complete blood count, blood urea nitrogen (BUN), serum creatinine, blood glucose, Na^+^, K^+^, SGOT, SGPT, alkaline phosphatase, bilirubin, T3, T4, TSH, CK-MB, and serial cardiac troponin I were all within normal ranges.

Treatment commenced with administration of furosemide, captopril, digitalis, and spironolactone.

After two days of his admission, he had an episode of bradycardia and respiratory apnea and was intubated due to low oxygen saturation and high Pa CO_2_ level.

The patient was intubated for 7 days without significant improvement in his condition, and in the 9th hospital day his blood pressure was not detected and cardiac rhythm became asystole; regardless of cardiopulmonary resuscitation for sixty minutes he remained pulseless and finally expired.

## 3. Discussion

Amyotrophic lateral sclerosis is the most well-known form of motor neuron diseases. ALS occurs in 1 to 2 people per 100,000. We presented an unusual case of amyotrophic lateral sclerosis that presented with cardiac failure at the final stage of the disease [1, 2].

Cardiac involvement has been rarely illustrated as part of the motor neuron diseases. Cardiac denervation related to involvement of the sympathetic nervous system has been depicted in patients in the early stages of ALS [3].

The current patient was not contented to undergo coronary angiography. So, due to high negative predictive value of myocardial perfusion scan for the absence of CAD in the setting of heart failure and left ventricular dysfunction, this noninvasive imaging modality was done for him [4].

The pathogenetic mechanism leading to an elevated occurrence of cardiomyopathy in ALS is not comprehensible. Dilated cardiomyopathy has been explained in some previous studies. Based on the collected data, it was hypothesized that cardiomyopathy is underdiagnosed in the ALS population, probably because symptoms are masqueraded as a result of the patients' disability. It was suggested that in all motor neuron diseases a serial cardiological evaluation should be executed, including annual echocardiography [5–7].

Moreover, the clinical impact of autonomic nervous dysfunction in ALS is vague in early stage but significant in the end stage of the disease, when ventilators are needed. Further studies may be required to show the pathognomonic importance of autonomic dysfunction in ALS [8].

## Figures and Tables

**Table 1:** Comparison of current echo parameters of the patient with 3-year-earlier echo findings.

Echo parameters	Time	
	3-year-earlier echo findings	Current echo findings
End-diastolic diameter	52.0 mm	66.0 mm
End-systolic diameter	37.0 mm	58.0 mm
Septal wall thickness	9.0 mm	10.0 mm
Lateral wall thickness	9.5 mm	10.0 mm
Ejection fraction	50%	20%
Left atrium area	17.5 cm^2^	32 cm^2^
Valvular abnormalities	Trivial MR, trivial TR	Moderate MR, mild TR
Diastolic dysfunction	Mild	Severe
